# Identification and targeting of CD22ΔE12 as a molecular RNAi target to overcome drug resistance in high-risk B-lineage leukemias and lymphomas

**DOI:** 10.20517/cdr.2017.03

**Published:** 2018-03-19

**Authors:** Fatih M. Uckun, Sanjive Qazi

**Affiliations:** 1 AresMIT Biomedical Computational Strategies (ABCS), Minneapolis, MN 55402, USA.; 2 Ares Pharmaceuticals, LLC, St. Paul, MN 55110, USA.; 3 Division of Hematology-Oncology, Department of Pediatrics, University of Southern California Keck School of Medicine (USC KSOM), Los Angeles, CA 90027, USA.; 4 Norris Comprehensive Cancer Center, University of Southern California Keck School of Medicine (USC KSOM), Los Angeles, CA 90027, USA.; 5 Bioinformatics Program, Gustavus Adolphus College, St. Peter, MN 56082, USA.

**Keywords:** Cancer, leukemia, RNA interference, nanomedicine, personalized medicine, driver lesion

## Abstract

**Aim:**

CD22ΔE12 as an oncogenic driver lesion in aggressive and drug-resistant B-precursor acute lymphoblastic leukemia (BPL) cells. The purpose of the present study was to identify the CD22ΔE12-specific signature transcriptome in human BPL cells and evaluate the clinical potential of a nanoscale formulation of CD22ΔE12-siRNA as an RNAi therapeutic against drug-resistant BPL. CD22ΔE12-siRNA nanoparticles significantly improved the event-free survival (EFS) outcome of NOD/SCID (NS) mice challenged with human BPL xenograft cells.

**Methods:**

Gene expression and translational bioinformatics methods were applied to examine the expression of the CD22ΔE12-specific signature transcriptome in human BPL cells in subsets of BPL patients. Survival analysis for mice challenged with BPL cells and treated with CD22ΔE12 siRNA was performed using standard methods.

**Results:**

Leukemia cells from CD22ΔE12-Tg mice exhibit gene and protein expression profiles consistent with constitutive activation of multiple signaling networks, mimicking the profiles of relapsed BPL patients as well as newly diagnosed high-risk patients with BCR-ABL^+^/Philadelphia chromosome (Ph)^+^ BPL as well as Ph-like BPL. A nanoscale formulation of CD22ΔE12-siRNA abrogated the *in vivo* clonogenicity of the leukemia-initiating leukemic cell fraction in xenograft specimens derived from patients with relapsed BPL and significantly improved the EFS outcome of NS mice challenged with drug-resistant human BPL xenograft cells.

**Conclusion:**

The CD22-RNAi technology is applicable to all BPL patients both high risk and standard risk. That is because CD22ΔE12 is a characteristic feature of drug-resistant leukemic clones that escape chemotherapy and cause relapse in both high risk and low risk subgroups of patients. The technology therefore has the potential (1) for prevention of relapses by selectively killing the clones that are most likely to escape chemotherapy and cause relapse as well (2) for treatment of relapses in BPL. This research project may also lead to innovative salvage regimens against other forms of CD22ΔE12-positive relapsed B-lineage leukemias and lymphomas.

## INTRODUCTION

B-precursor acute lymphoblastic leukemia (BPL) is the most common form of cancer in children and adolescents and a leading cause of cancer-related mortality in children^[1–5]^. Currently, the major challenge in the treatment of BPL is to cure patients who have relapsed despite intensive frontline chemotherapy^[2–4]^. There is an unmet and urgent medical need for identification of new agents capable of killing chemotherapy (“drug”)-resistant BPL cells or selectively augmenting their drug sensitivity. The identification of new molecular targets and new treatment strategies that can help overcome the drug resistance of BPL cells would be an important step forward in efforts aimed at improving the treatment outcomes of relapsed BPL patients.

Hogan *et al.*^[6]^ reported the results of an integrated genomic analysis of primary leukemia cells from relapsed BPL patients, which showed that relapse clones are characterized by concomitant activation of mitogen-activated protein kinase (MAPK) and WNT pathways - but the driver lesion that causes this distinct gene expression signature has remained elusive. Both pathways have been implicated in chemotherapy resistance and aggressiveness of BPL cells. Likewise, constitutive activation of the phosphatidylinositol 3-kinase (PI3-K), Akt and the mammalian target of rapamycin (mTOR) (PI3K/Akt/mTOR) network is a characteristic feature of aggressive BPL cells. BPL cells express a dysfunctional CD22 due to deletion of exon 12 (CD22ΔE12) arising from a splicing defect associated with homozygous intronic mutations^[7]^. CD22ΔE12 results in a truncating frame shift mutation yielding a mutant CD22ΔE12 protein that lacks most of the intracellular domain including the key regulatory signal transduction elements and all of the cytoplasmic tyrosine residues. CD22ΔE12 is a driver lesion that we recently identified as the likely genetic cause for the drug resistance-associated activation of MAPK, PI3-K/m-TOR and WNT pathways in primary leukemia cells from relapsed as well as newly diagnosed high-risk BPL patients (including the BCR-ABL^+^/Ph^+^ subset)^[7,8]^. Our recent studies have provided direct evidence that CD22ΔE12 is a characteristic genetic defect of therapy-refractory clones in pediatric BPL and implicated the CD22ΔE12 genetic defect in the aggressive biology of relapsed or therapy-refractory pediatric BPL^[8]^. More recent studies revealed a very high incidence of CD22ΔE12 in both pediatric and adult aggressive B-lineage lymphoid malignancies^[9,10]^. Examination of the CD22ΔE12 index in genetically defined high-risk BPL patient subsets revealed a high incidence of CD22ΔE12 in primary BPL cells from newly diagnosed high-risk BPL patients as well^[9–11]^. Our studies using quantitative real time RT-PCR on samples obtained from 114 newly diagnosed pediatric ALL patients showed a high incidence of CD22ΔE12 in pediatric BPL and confirmed the presence of this genetic defect in 100% (14/14) of infant ALL cases^[7–11]^.

Notably, forced expression of the mutant human CD22ΔE12 protein in transgenic (Tg) mice under control of the immunoglobulin enhancer Eμ that is activated in early B-cell ontogeny prior to immunoglobulin gene rearrangements caused fatal CD19^+^CD24^+^CD45R/B220^+^CD127/IL7-R^+^sIgM^−^ BPL in C57/BL/6 mice^[11]^. This Tg mouse model recapitulates the gene expression profile of CD22ΔE12^+^ human BPL, establishing a causal relationship between CD22ΔE12 and BPL and indicating that CD22ΔE12 alone as a driver lesion is sufficient for malignant transformation and clonal expansion of B-cell precursors^[11]^. BPL cells from CD22ΔE12-Tg mice exhibit characteristic gene expression and protein expression profiles consistent with constitutive activation of multiple signaling networks, mimicking the profiles of relapsed BPL patients^[7–11]^.

Functional RNA interference experiments using CD22ΔE12-specific siRNA and its nanoscale formulations have confirmed the causal link between CD22ΔE12 and the stemness features as well as aggressiveness and chemotherapy resistance of leukemic B-cell precursors from BPL patients^[8–11]^. CD22ΔE12 depletion by CD22ΔE12-specific small interfering RNA (siRNA) caused a loss of the *in vitro* clonogenicity of BPL xenograft cells in colony forming assays and abrogated their *in vivo* clonogenic, leukemia initiating and propagating properties in immunodeficient NOD/SCID mice^[8–11]^. The purpose of the present study was to identify the CD22ΔE12-specific signature transcriptome in human BPL cells and further evaluate the clinical potential of a nanoscale formulation of CD22ΔE12-siRNA as an RNAi therapeutic against drug-resistant BPL. CD22ΔE12-siRNA nanoparticles significantly improved the event-free survival (EFS) outcome of NOD/SCID (NS) mice challenged with human BPL xenograft cells.

## METHODS

### Leukemia cells

We used BPL xenograft clones (*n* = 2) that were derived from spleen specimens of xenografted NS mice inoculated with human BPL cells from deidentified left-over fresh bone marrow specimens from pediatric BPL patients. The secondary use of leukemic cells for subsequent laboratory studies did not meet the definition of human subject research per 45 CFR 46.102 (d and f) since it did not include identifiable private information, and it was approved by the IRB (CCI) at the Children’s Hospital Los Angeles (CHLA).

### Animal research approval

The animal research in mice was conducted according to Institutional Animal Care and Use Committee (IACUC) Protocols 280–12 and 293–10 that were approved by the IACUC of CHLA. All animal care procedures conformed to the Guide for the Care and Use of Laboratory Animals (National Research Council, National Academy Press, Washington DC 1996, USA).

### Preparation and characterization of the liposomal CD22ΔE12 siRNA formulation

In order to achieve RNAi in all leukemic cells in a targeted cell population *in vitro* and for *in vivo* RNAi experiments, we prepared a liposomal nanoformulation (LNF) of the CD22ΔE12 siRNA duplex with an encapsulation efficiency of 96.3% ± 0.7% using the standard thin film evaporation method in a round bottom flask^[11]^. We used a mixture of the cationic lipid 2,3-dioleoyloxypropyltrimethylammonium chloride (DOTAP; Cas Number: 132172–61-3) (3.9 mmol/L) and the neutral lipid 1,2-dioleoyl-sn-glcero-3-phosphoethanolamine (DOPE; Cas Number: 4004–5-1) (3.9 mmol/L). Both lipids were purchased from Avanti Polar Lipids (Alabaster, Alabama). siRNA-free (formulation 4B) and scr-siRNA loaded (formulation 4C) control liposomal formulations were also prepared for comparison. We used the Quant-IT RiboGreen RNA assay (Invitrogen) and a Synergy HT Biotek fluorescence microplate reader to measure the siRNA content of the liposomal formulations in the presence and absence of 1% TritonX100 for “burst-release” of their siRNA content. The presence of siRNA in the formulations was also confirmed in 2% agarose gels prepared using 0.5 μg/mL of ethidium bromide and 3% SDS that allows staining of the siRNA content with ethidium bromide^[11]^. Size measurement by the dynamic light scattering (DLS) technique was performed on a DynaPro Titan Instrument (Wyatt Technology Corp., Santa Barbara, CA) at the USC Nano BioPhysics Laboratory. The Zeta potential measurements were carried out using a Zetasizer Ver 6.12 (Serial #MAL1044603) (Malvern Instruments, UK). The generated liposomal CD22ΔE12-siRNA formulation had a radius of 132.5 ± 4.7 nm, a positive surface charge with a Zeta potential of 54.8 mV in solution consistent with the use of positively charged DOTAP and contained 33 ± 2 μmol/L CD22ΔE12-siRNA^[11]^.

### Survival analysis for mice treated with CD22ΔE12 siRNA

NS mice were inoculated i.v. with xenograft cells (4 × 10^5^ cells/mouse) derived from primary leukemia cells of 2 pediatric patients with BPL^[11]^. The EFS curves were generated using the Kaplan-Meier product limit method^[11]^. Sixteen control mice were either left untreated or treated with the LNF of scr-siRNA (25 nmol/kg per day × 3 days, days 1–3) or an empty control LNF. Test mice were treated with the CD22ΔE12-siRNA LNF (low-dose regimen = 2.5 nmol/kg per day × 3 days, days 1–3, *n* = 10; high-dose regimen = 25 nmol/kg per day × 3 days, days 1–3, *n* = 9). Box plots depicted time to event and time to censor. Proportion of mice remaining alive for control were compared to CD22ΔE12-siRNA LNF treated (pooled low and high doses) mice and the median time to event were compared using Wilcoxon non-parametric test for the 2 groups of mice.

### Gene expression profiling of murine BPL cells

Gene expression values for samples obtained from WT (*n* = 4) and transgenic mice (*n* = 2 for CD22ΔE12-Tg, Eμ-MYC Tg, BCR-ABL Tg) were estimated from Robust Microarray Analysis (RMA) normalization of signal values following hybridization to the Affymetrix Mouse Gene 1.0 ST Array (1,102,500 probes, 35,512 genes). Perfect Match (PM) signal values for probesets were extracted utilizing raw CEL files matched with probe identifiers obtained from a CDF file (MoGene-1_0-st-v1,r3.cdf) implemented by Aroma Affymetrix statistical packages ran in R-studio environment (Version 0.97.551, R-studio Inc., running with R 3.01). The PM signals were quantified using Robust Multiarray Analysis in a 3-step process including RMA background correction, quantile normalization, and summarization by a log-additive model of probes in a probeset across 14 samples (RmaPlm method adapted in Aroma Affymetrix). All expression values were log_2_ scaled. The relative log expression (RLE) and normalized unscaled standard error (NUSE) plots were utilized to assess array quality. For each gene and each array, ratios were calculated between the expression of a gene and the median expression of this gene across all arrays of the experiment. Box plot of the RLE and NUSE were within the quality bounds for all the arrays. *T*-values greater than 5 (2035 genes) or less than −5 (1973 genes) of log2 expression difference between CD22ΔE12-Tg mice (*n* = 2) pooled WT and Eμ-MYC Tg or BCR-ABL Tg mice (*n* = 8) were visualized using a one-way hierarchical cluster figure to organize similar expression profiles of genes across the samples.

Transcript cluster annotations for the mouse array were obtained from NetAffx website (http://www.affymetrix.com/Auth/analysis/downloads/na33/wtgene-32_2/MoGene-1_0-st-v1.na33.2.mm9.transcript. csv.zip). *T*-values of the difference between CD22ΔE12-Tg and WT plus Eμ-MYC Tg or BCR-ABL Tg mice were processed for Gene Set Enrichment Analysis (GSEA) in 665 gene sets obtained from the Reactome Database (c2.cp.reactome.v4.0.symbols deposited in database on broadinstitute.org servers) using a supervised approach implemented in GSEA2.08 (Broad institute). Significance of association was assessed using weighted Kolmogorov-Smirnov statistics. GSEA evaluated significance of the over-representation of probesets differentially expressed in CD22ΔE12-Tg mice by calculating the Enrichment Score (ES) representing the difference between the observed rankings from the expected null. The null distribution assumed a random rank distribution utilizing an empirical permutation test procedure that randomly assigned probeset names to the rank ordered differences in expression (“GSEA Pre-ranked” algorithm). Leading edge genes were identified up to and including the peak of the ES profile. Nominal *P*-values were computed by comparing the tails of the ES scores for observed and permutation-generated null distributions following 1000 permutations. Following this procedure, 316 gene sets were significantly enriched at nominal *P*-value < 5% and 277 gene sets were significantly enriched at nominal *P*-value < 1% for genes upregulated in CD22ΔE12-Tg mice.

### Phosphoproteome analysis using antibody microarrays

The Phospho Explorer Antibody Array (Full Moon BioSystems, Inc., Sunnyvale, CA) consists of 1330 duplicate spots interrogating 1318 proteins (phosphorylated and unphosphorylated), house-keeping proteins (Beta actin, GAPDH), negative controls (*n* = 4), empty spots (*n* = 4) and positive markers (*n* = 2)^[10,11]^. The antibodies are printed on standard-size coated glass slides and can be scanned on all microarray scanners that are compatible with 76 mm × 25 mm × 1 mm (3 inch × 1 inch × 1 mm) slides. The antibody array analyses were performed on cell lysate protein samples according to standard protocols. In brief, the cell lysates were biotinylated for 2 h at room temperature using the Biotin reagent in the antibody array assay kit (Full Moon BioSystems, Inc.). The array slide was blocked with blocking solution for 30 min at room temperature on an orbital shaker and then rinsed 10 times with Milli-Q grade water. The biotinylated proteins (~80 μg) were mixed with 6 mL of coupling solution (Full Moon Biosystems, Inc.) and then added over the array slide for a 2-h incubation at room temperature while shaking. The array was then washed with 30 mL of 1 × Wash Solution (Full Moon Biosystems, Inc.) for 10 min for a total of three times and then rinsed ten times with Milli-Q water before detection of bound biotinylated proteins using Cy3-conjugated streptavidin. Subsequently, the array slide was incubated in Cy3-Streptavidin solution for 30 min in the dark while shaking. Next, the slide was washed with 1 × Wash buffer and rinsed with Milli-Q water as before. The slide was then dried with compressed nitrogen and scanned using Axon GenePix 4000 scanner and the images were analyzed with GenePix Pro 6.0 (Molecular Devices, Sunnyvale, CA). The fluorescence signal of each antibody was obtained from the fluorescence intensity of this antibody spot after subtraction of the blank signal (spot in the absence of antibody). Two technical replicates were performed for each sample. Median signal intensity for each spot was extracted from array images and the average intensity for each antibody reaction with the protein was determined for replicate spots. Data were normalized utilizing the median intensity values for all the spots on each array (normalized data = average signal intensity of replicate spots/median signal). The normalized data were log_10_ transformed and mean centered to the WT samples (*n* = 4; 2 technical replicates for each of 2 samples) (GSE58873 and GSE58874). We extended our previous study to characterize a robust gene signature for CD22ΔE12^[10]^. A Nested ANOVA model was utilized to identify significantly regulated gene products in CD22ΔE12 expressing mice comparing Wild Type (*n* = 4), CD22ΔE12-Tg (*n* = 9) and CD22ΔE12-knock in (KI)^[9]^ (*n* = 5) samples. Factors for the model included: ProteinID, Group, ProteinID × Group, replicate nested within Group. Linear contrasts were performed comparing CD22ΔE12-Tg *vs.* WT and CD22ΔE12-KI *vs.* WT resulting in identification of 86 proteins with *P* < 0.001 for both comparisons.

### Gene expression profiling of murine BPL cells

Murine BPL cells were isolated from markedly enlarged spleens of CD22ΔE12-KI or CD22ΔE12-Tg mice, whereas splenocytes from WT healthy C57BL/6 mice served as controls. Total cellular RNA was extracted from homogenized lysate samples of leukemia cells using the Qiagen RNeasy Plus Mini Kit (Cat No 74134) (Qiagen, Santa Clarita, CA). In order to minimize the genomic DNA contamination in the RNA samples, the homogenized lysate samples were first run through a DNA binding spin column prior to steps of RNA binding, washing and elution. Total RNA (45 μL/sample) was obtained with a concentration range of 60–200 ng/mL and an absorbance ratio (A260/A280) ranging from 1.9–2.1. RNA quality as judged by the integrity of the 28S and 18S ribosomal RNA was determined by an Agilent 2100 Bioanalyzer to ensure that it was acceptable for subsequent microarray analysis. Gene expression values for splenocytes from WT healthy C57/BL/6 mice (*n* = 4), leukemia cells from CD22ΔE12-Tg mice (*n* = 6), leukemia cells from CD22ΔE12-KI mice (*n* = 6) were estimated from RMA normalization of signal values following hybridization to the Affymetrix Mouse Gene 1.0 ST Array (1,102,500 probes, 35,512 genes) (GSE58874 and GSE58872). PM signal values for probesets were extracted utilizing raw CEL files matched with probe identifiers obtained from a CDF file (MoGene-1_0-st-v1,r3.cdf obtained from http://www.aroma-project.org/vignettes/GeneSTArrayAnalysis) implemented by Aroma Affymetrix statistical packages run in the R-studio environment (Version 0.97.551, R-studio Inc., running with R 3.01). The PM signals were quantified using RMA in a 3-step process including RMA background correction, quantile normalization, and summarization by a log additive model of probes in a probeset across these samples (RmaPlm method adapted in Aroma Affymetrix). All expression values were log_2_ scaled. The RLE and NUSE plots were utilized to assess array quality. For each gene and each array, ratios were calculated between the expression of a gene and the median expression of this gene across all arrays of the experiment. Box plots of the RLE and NUSE were within the quality bounds for all the arrays. Expression changes were visualized using a one-way hierarchical cluster figure to organize similar expression profiles of genes across the samples.

### CD22ΔE12-driven gene expression cassette

Eighty-six significantly upregulated phosphoproteins in CD22ΔE12-Tg and CD22ΔE12-KI mice (*P* < 0.001) were represented by 71 mouse genes (74 probesets) identified by cross referencing the Swiss Prot ID provided by the manufacturers of the antibody array with the Affymetrix mogene10 annotation data provided by Bioconductor for R [mogene10sttranscriptcluster.db file as well as the conversion tool provided by MGI, Jackson Laboratory (http://www.informatics.jax.org/homology.shtml)]. These mouse probesets were compared for differential expression in 6 CD22ΔE12-Tg samples or 6 CD22ΔE12-KI samples *vs.* 4 WT samples utilizing a Mixed Model ANOVA (lme4 package from R; 2 fixed factors for “probeset” and “genotype” (CD22ΔE12 and WT), 1 interaction term for “probeset × genotype”, and 1 random factor for mouse ID) to identify significantly affected mRNA levels (least square means and standard error estimates calculated from the interaction term parameters) for the significantly affected individual probesets and significance of the mouse gene signature as a whole utilizing the least mean squares and standard error estimates obtained from the “genotype” factor.

We compiled archived transcriptome profiling datasets in the Human Genome U133 Plus 2.0 Array platform regarding gene expression levels in primary leukemia cells from pediatric BPL patients (GSE11877, *n* = 207; GSE13159, *n* = 823; GSE13351, *n* = 107; GSE18497, *n* = 82, GSE28460, *n* = 98; GSE7440, *n* = 99; total number in 6 datasets = 1416). To enable comparison of samples across studies, a normalization procedure was performed that merged the raw data from the 6 datasets (CEL files) for the pediatric patients. Perfect Match (PM) signal values for probesets were extracted utilizing raw CEL files matched with probe identifiers obtained from the Affymetrix provided CDF file (HG-U133_Plus _ 2.cdf) implemented by Aroma Affymetrix statistical packages ran in R-studio environment (Version 0.97.551, R-studio Inc., running with R 3.01). The PM signals were quantified using RMA in a 3-step process including RMA background correction, quantile normalization, and summarization by Median Polish of probes in a probeset across 1416 pediatric (RMA method adapted in Aroma Affymetrix). RMA background correction estimates the background by a mixture model whereby the background signals were assumed to be normally distributed and the true signals are exponentially distributed. Normalization was achieved using a two-pass procedure. First the empirical target distribution was estimated by averaging the (ordered) signals over all arrays, followed by normalization of each array toward this target distribution.

In order to examine the representation of this CD22ΔE12-dictated gene expression signature in human BPL cells, we first identified the human orthologs on the Affymetrix Human Genome U133 Plus 2.0 Array (hgu133plus2.db file downloaded from the Bioconductor repository Affymetrix by cross-referencing the hugene10 annotation data (hugene10sttranscriptcluster.db file) provided by the Bioconductor repository for R software (http://www.bioconductor.org/) as well as the conversion tool provided by MGI, Jackson Laboratory (http://www.informatics.jax.org/homology.shtml). Mixed Model ANOVAs (2 fixed factors for “probeset” and “diagnostic group”, 1 interaction term for “probeset × diagnostic group” and 1 random factor for sample ID) were constructed for each comparison to assess probeset level significance (least square means and standard error estimates obtained from the interaction term parameters) and significance of the mouse gene signature as a whole for human leukemic group comparisons (least mean squares and standard error estimates obtained from the “diagnostic group” factor).

### Identification of Ph-like subset of BPL patients

Ph-like cases were previously identified using a set of 110-probeset gene expression signature developed utilizing the Affymetrix U133 plus 2.0 microarray platform^[12,13]^. In our study, expression values for each of the 110 probesets were averaged across 123 BCR-ABL positive cases from the normalized database of 1416 patients to provide an expression template for subsequent correlation analysis. Three studies (GSE11877, GSE13159, GSE13351) referenced normal (*n* = 74), T-cell ALL (*n* = 189), BCR-ABL^+^/Ph^+^ BPL (*n* = 123), E2APBX1^+^ BPL (*n* = 61), MLL re-arrangement (R)^+^ BPL (*n* = 95) and other (*n* = 595) subtypes of primary samples. Gene expression of each sample was correlated against the 110 probeset expression template resulting in a distribution of correlation coefficient values (*r*) for each subtype of primary samples. Ph-like samples were defined from a cut off “*r*” value such that the sample from the “*r*” value was greater than the 99% percentile of the “*r*” values in normal samples and greater than the minimum “*r*” value for the known Ph^+^ samples. The “other” group was further partitioned into “Ph-like” and “other” groups based on the “*r*” value cut off.

### Significantly affected mouse gene signatures representation in human BPL

We investigated the phosphoproteome (86 phosphoproteins)-validated signature genes (74 probesets/71 genes for their expression in BCR-ABL/Ph^+^ BPL (*n* = 123; GSE13159, GSE13351) *vs.* other BPL (*n* = 225; GSE11877, GSE 13159, GSE13351), Ph-like BPL (*n* = 370; GSE11877, GSE13159, GSE13351) *vs*. other BPL (*n* = 225) and BPL in relapse (*n* = 76; GSE18497, GSE28460) *vs*. newly diagnosed BPL (*n* = 474; GSE11877, GSE13351, GSE18497, GSE28460, GSE7440) patients. Mixed Model ANOVA were used to determine significance of individual probesets and overall changes in the expression of the gene set as a whole in mice and human BPL.

### Determination of CD22ΔE12-index values by multiprobe gene expre ssion profiling

BLAT analysis on CD22 probe sequences for probeset 217422_s_at (covering exons 10–14) deposited in the Affymetrix NetAffx™ Analysis Center (http://www.affymetrix.com/analysis/index.affx) were mapped onto specific CD22 exons and visualized using the UCSC genome browser (http://genome.ucsc.edu/cgi-bin/hgBlat?command=start). This analysis was designed to locate ≥ 25-bp long sequences with ≥ 95% similarity in the entire genome. The BLAT-based exon designations according to 3 reference sequences (viz.: UCSC genes, Ensembl gene predictions, Human mRNA Genbank) were as follows: HG-U133_PLUS_2:217422_S_AT_11 aligned to chr19: 35837525–35837549 (Exon 14); HG-U133_PLUS_2:217422_S_AT_10 aligned to chr19: 35837478–35837502 (Exon 14); HG-U133_PLUS_2:217422_S_AT_8 aligned to chr19: 35837100–35837124 (Exon 13); HG-U133_PLUS_2:217422_S_AT_9 aligned to chr19: 35837117–35837139 (Exon 13); HG-U133_PLUS_2:217422_S_AT_7 aligned to chr19:35836590–35836614 (Exon 12); HG-U133_PLUS_2:217422_S_AT_6 aligned to chr19:35836566–35836590 (Exon 12); HG-U133_PLUS_2:217422_S_AT_5 aligned to chr19:35836535–35836559 (Exon 12); HG-U133_PLUS_2:217422_S_AT_4 aligned to chr19:35835979–35836003 (Exon 11); HG-U133_PLUS_2:217422_S_AT_3 aligned to chr19:35835811–35835960 (Exon 11); HG-U133_PLUS_2:217422_S_AT_2 aligned to chr19:35835741–35835765 (Exon 10). Background corrected and RMA-normalized signal values for each probe in each sample were log_2_ transformed and median centered across 11 probes per sample. An Exon 12 Index was calculated by subtracting the median centered expression values of the Exons 10–11 plus Exons 13–14 probes from the median centered expression values of the Exon 12 probes. The CD22ΔE12 index values were calculated and compared for normal bone marrow hematopoietic cells *vs*. T-cell ALL (*n* = 189; GSE13159 and GSE13351) *vs*. MLL-R^+^ BPL (*n* = 95; GSE11877, GSE13159, GSE13351), BCR-ABL/Ph^+^ BPL (*n* = 123; GSE13159, GSE13351) *vs*. E2A-PBX1^+^ BPL (*n* = 61; GSE11877, GSE13159, GSE13351) *vs*. Ph-like BPL (*n* = 370; GSE11877, GSE13159, GSE13351). To compare the incidence of CD22ΔE12 in subsets of patients the proportion of leukemic samples below the lower 95% confidence interval for normal non-leukemic cells *vs*. leukemia cells for each patient sub-population was determined. Planned linear contrasts derived from one-way ANOVA were utilized to calculate *P*-values of the comparisons.

## RESULTS

### Signature transcriptome and proteome of CD22ΔE12-Tg mouse BPL cells

**Figure 1.**
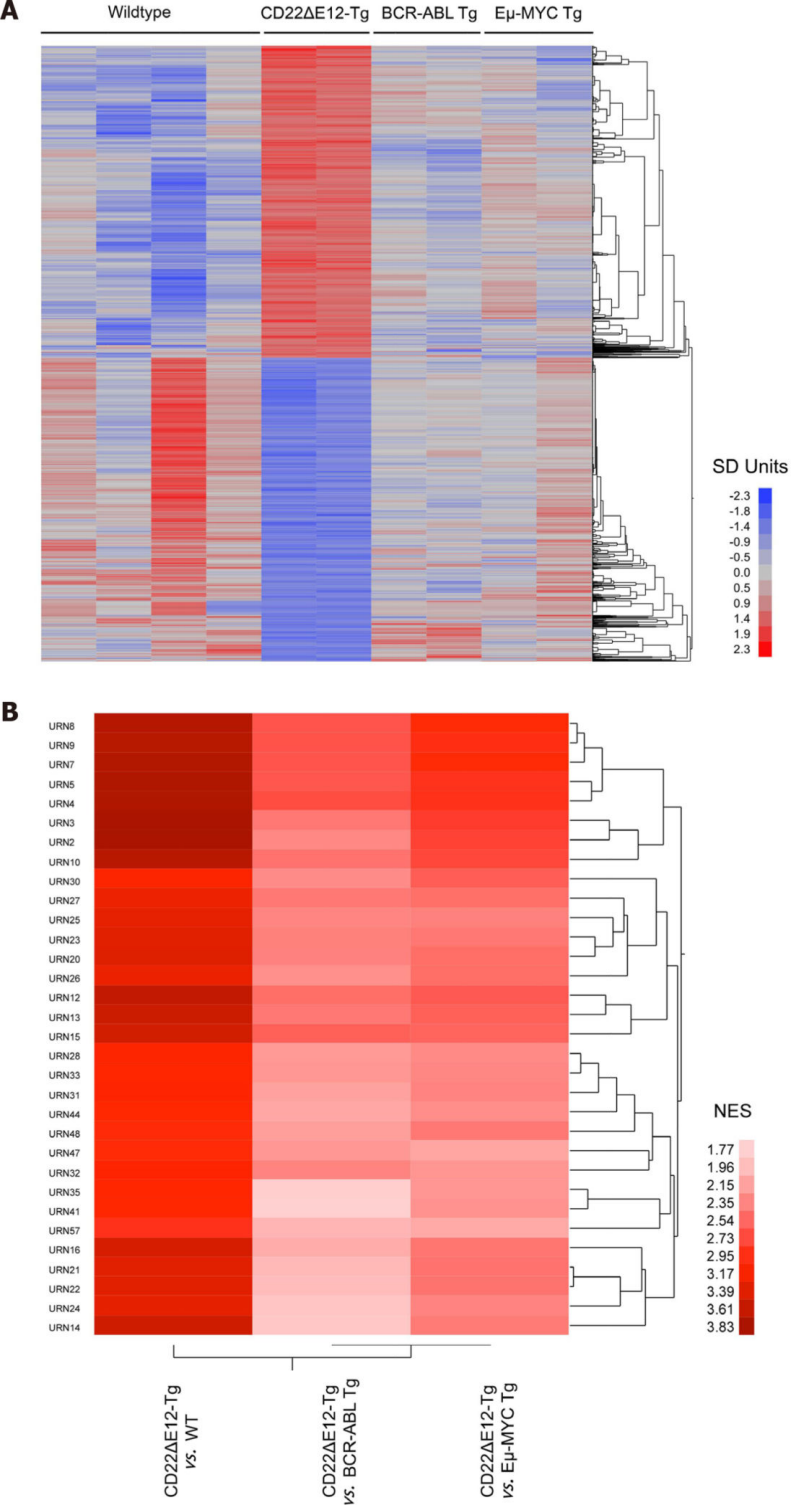
Unique signature transcriptome of CD22ΔE12 transgenic (Tg) BPL cells. (A) Depicted is a one-way hierarchical cluster figure that illustrates gene expression differences between BPL cells from CD22ΔE12-Tg mice and normal splenocytes from healthy WT mice, BPL cells from BCR-ABL Tg mice or BPL cells from Eμ-MYC Tg mice; (B) Depicted is a two-way agglomerative hierarchical cluster figure whereby the dendrograms were joined by the average distance metric. The heat map depicts the NES scores for 32 Reactome pathway gene sets related to DNA replication, transcription, translation and cell cycle regulation that showed significant upregulation (*P* < 0.01) for all three comparisons of CD22ΔE12-Tg *vs*. WT, CD22ΔE12-Tg *vs*. Eμ-MYC Tg and CD22ΔE12-Tg *vs*. BCR-ABL Tg. A sub-cluster of highly significant enrichment of 7 large Reactome pathways (URN 2, 3, 4, 5, 7, 8, and 9 - see Supplementary Table 1) (*P* < 0.001 for all comparisons) was revealed in this representation highlighting the role of CD22ΔE12 in cellular re-programming

Gene expression profiling revealed differential upregulation of MAPK, PI3-K/mTOR, WNT, and JAK/STAT pathway genes in BPL cells from CD22ΔE12-Tg mice closely mimicking the transcriptome of primary leukemia cells from high-risk BPL patients (Accession #GSE58872 and GSE58874) [Figure 1]. Comparison of log_2_ gene expression differences between BPL cells from CD22ΔE12 Tg mice (*n* = 2) *vs*. Other cells (*n* = 8; pooled data on wildtype normal splenocytes and BPL cells from BCR-ABL-Tg or Eμ-MYC Tg mice) identified 8004 probsets that were downregulated and 5443 probesets that were upregulated with *P* < 0.05). More specific pairwise comparisons revealed that out of the 35,511 probesets that were evaluated, 3656 (1999 down regulated and 1657 upregulated), 2608 (1491 downregulated and 1117 upregulated) and 10,644 probesets (5901 down regulated and 4753 upregulated) were differentially expressed with *P* < 0.05 in CD22ΔE12-Tg mice compared to BCR-ABL Tg mice, Eμ-MYC Tg mice and WT mice respectively. We further examined these expression changes using gene set enrichment analysis in gene sets obtained from the Reactome Database (c2. cp.reactome.v4.0.symbols deposited in database on broadinstitute.org servers) using a supervised approach implemented in GSEA2.08 (Broad institute). Normalized enrichment scores demonstrated a CD22ΔE12-induced widespread dysregulation of gene expression levels compared to WT (324 and 52 gene sets exhibited enrichment in up and down regulated expression in CD22ΔE12-Tg mice respectively with *P* < 0.05; 287 and 27 gene sets exhibited enrichment in up and down regulated expression respectively in CD22ΔE12-Tg mice with *P* < 0.01), BCR-ABL Tg (192 and 39 gene sets exhibited enrichment in up and down regulated expression respectively in CD22ΔE12-Tg mice with *P* < 0.05; 130 and 12 gene sets exhibited enrichment in up and down regulated expression respectively in CD22ΔE12 Tg mice with *P* < 0.01) and Eμ-MYC Tg mice (286 and 9 gene sets exhibited enrichment in up and down regulated expression respectively in CD22ΔE12-Tg mice with *P* < 0.05; 163 and 3 gene sets exhibited enrichment in up and down regulated expression respectively in CD22ΔE12-Tg mice with *P* < 0.01). One hundred and seventy-eight gene sets exhibited enrichment in up regulated probesets across all 3 comparisons (*P* < 0.05; CD22ΔE12-Tg mice compared to BCR-ABL Tg mice, Eμ-MYC Tg mice and WT mice). Cluster analysis of highly enriched gene sets revealed that transcriptional, translational and cell cycle processes were significantly upregulated in all three comparisons of CD22ΔE12 Tg mice compared to BCR-ABL Tg, Eμ-MYC Tg and WT mice [7 large Reactome pathways with *P* < 0.001 for all comparisons; REACTOME_3_UTR_MEDIATED_TRANSLATIONAL_REGULATION (URN8), REACTOME_PEPTIDE_CHAIN_ELONGATION (URN9), REACTOME_NONSENSE_MEDIATED_DECAY_ENHANCED_BY_THE_EXON_JUNCTION_COMPLEX (URN7), REACTOME_SRP_DEPENDENT_COTRANSLATIONAL_PROTEIN_TARGETING_TO_MEMBRANE (URN5), REACTOME_TRANSLATION (URN4), REACTOME_CELL_CYCLE_MITOTIC (URN3), and REACTOME_DNA_REPLICATION (URN2)]. The GSEA-NES values, nominal *P*-values and URN designation for the interrogated Reactome pathways, including the 32 genesets shown in Figure 1, are depicted in Supplementary Table 1. The top 10 leading edge probesets and the human homolog gene symbols with the corresponding *T*-value are shown in Supplementary Table 2. URN 2: REACTOME_DNA_REPLICATION; URN 3: REACTOME_CELL_CYCLE_MITOTIC; URN 4: REACTOME_TRANSLATION; URN 5: REACTOME_SRP_DEPENDENT_COTRANSLATIONAL_PROTEIN_TARGETING_TO_MEMBRANE; URN 7:REACTOME_NONSENSE_MEDIATED_DECAY_ENHANCED_BY_THE_EXON_JUNCTION_COMPLEX; URN 8: REACTOME_3_UTR_MEDIATED_TRANSLATIONAL_REGULATION; URN 9: REACTOME_PEPTIDE_CHAIN_ELONGATION.

Our previous studies identified 34 phosphoproteins were most significantly over expressed in CD22ΔE12-Tg mice^[10]^. These proteins were represented by 32 genes on the Mouse Gene 1.0 ST oligonucleotide array platform that examined mRNA levels in CD22ΔE12-Tg mice, of these 10 genes exhibited significant increases in expression in these mice. Furthermore, 8 out of the 10 genes (*ATF1*, *ATF2*, *MTOR*, *PLCG2*, *PTK2*, *RELA*, *RPS6KB1* and *TSC2*) were also over expressed in 76 relapsed cases. The present study extends these findings using increased sample size for CD22ΔE12-Tg and developed a gene signature that also included CD22ΔE12-KI mice. Mouse orthologs of the previously identified gene set in relapsed cases were significantly up regulated in CD22ΔE12-KI mice (Mixed Model ANOVA, F_1,8_ = 86.1, *P* < 0.0001; all mouse transcripts were up regulated). We now report that 86 phosphoproteins were significantly upregulated (*P* < 0.001) in both CD22ΔE12-Tg and CD22ΔE12-KI mice. These phosphoproteins were represented by 71 mouse genes (74 probesets) on the mouse gene chip. Examination of gene expression changes demonstrated that 57 (59 probesets ) out of the 71 genes were significantly upregulated in CD22ΔE12-KI mice (*P* < 0.01), representing a high concordance of protein expression and gene expression changes with additional genes identified in the present study.

**Figure 2.**
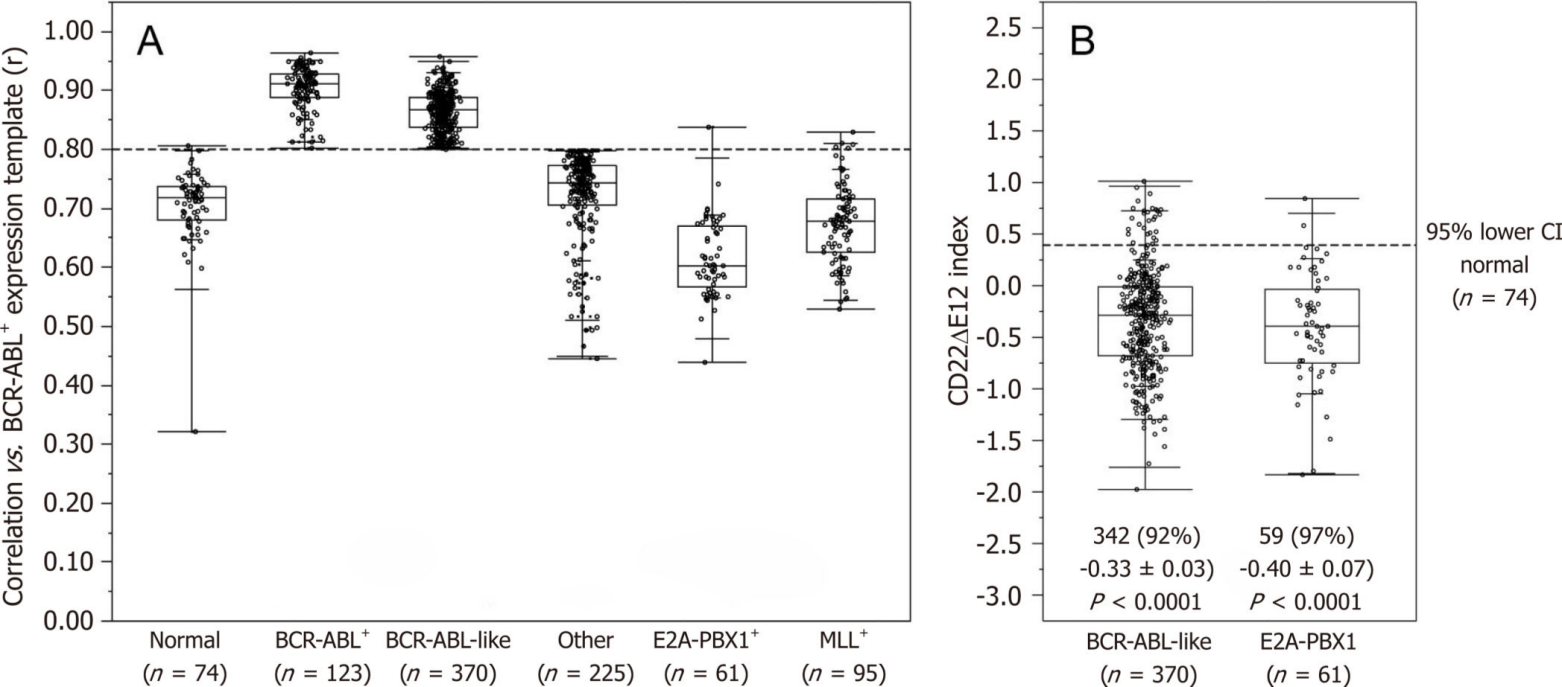
Incidence of CD22ΔE12 in BCR-ABL^+^/Ph^+^ and Ph-like ALL patients. (A) The expression values for each of the 110 probesets were averaged across 123 BCR-ABL^+^ (“Ph^+^”) cases from the normalized database of 1416 patients to provide an expression template for subsequent correlation analysis; (B) BLAT analysis on CD22 probe sequences for probeset 217422_s_at (covering exons10–14) deposited in the Affymetrix NetAffx™Analysis Center were mapped onto specific CD22 exons and visualized using the UCSC genome browser (http://genome.ucsc.edu/cgibin/hgBlat?command=start). A CD22ΔE12 Index was calculated by subtracting the median centered expression values of the 7 probes for CD22 Exons 10–11 and CD22 Exons 13–14 in the 217422_s_at probeset from the median centered expression values of the 3 CD22 Exon 12 probes. Depicted is the Quantile dot plot (Box represents 25th, median and 75th percentiles, and the whiskers represent the minimum, 10th, 90th and maximum values) of the sample level CD22ΔE12-index values in primary leukemic samples from 123 Ph^+^ and 370 Ph-like ALL patients

**Figure 3.**
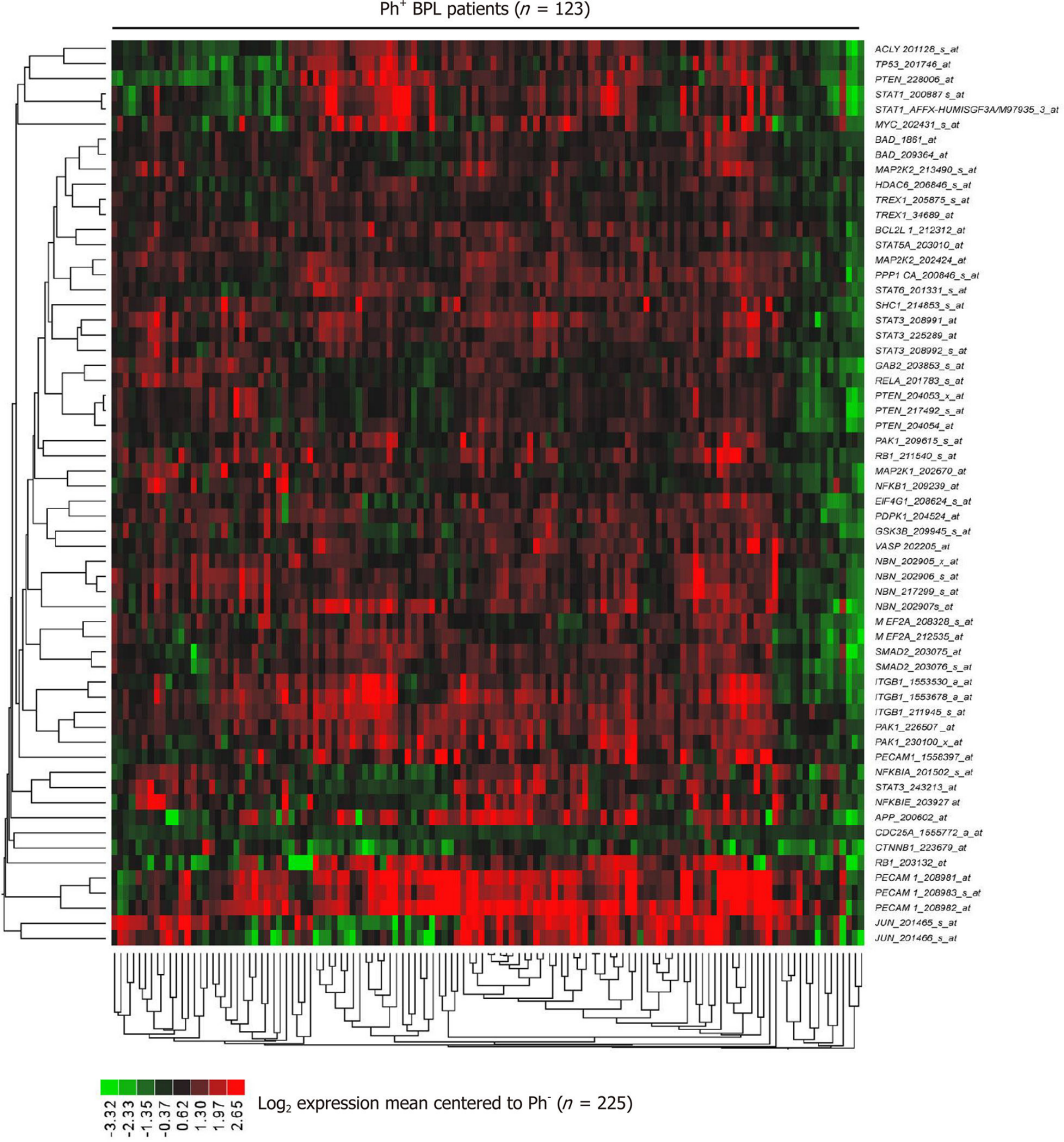
Expression of CD22ΔE12 signature genes in Ph^+^/BCR-ABL^+^ BPL. The RMA-normalized gene expression values for leukemia cells obtained from 123 BCR-ABL^+^ patients [GSE13159 (*n* = 122), GSE13351 (*n* = 1) were log_2_-transformed and mean-centered to the average value for leukemia cells from 225 BCR-ABL^−^ BPL patients (GSE1187, GSE13159, GSE13351; Ph-like ALL, MLL-R^+^ and t[1;19]/E2A-PBX1^+^ patients excluded)]. To determine the differential expression of each of the leading-edge genes of the CD22ΔE12 transcriptome, linear contrasts were performed for the mean-centered values. Heat map depicts up and down regulated transcripts for mean-centered expression values and clustered according to average distance metric

**Figure 4.**
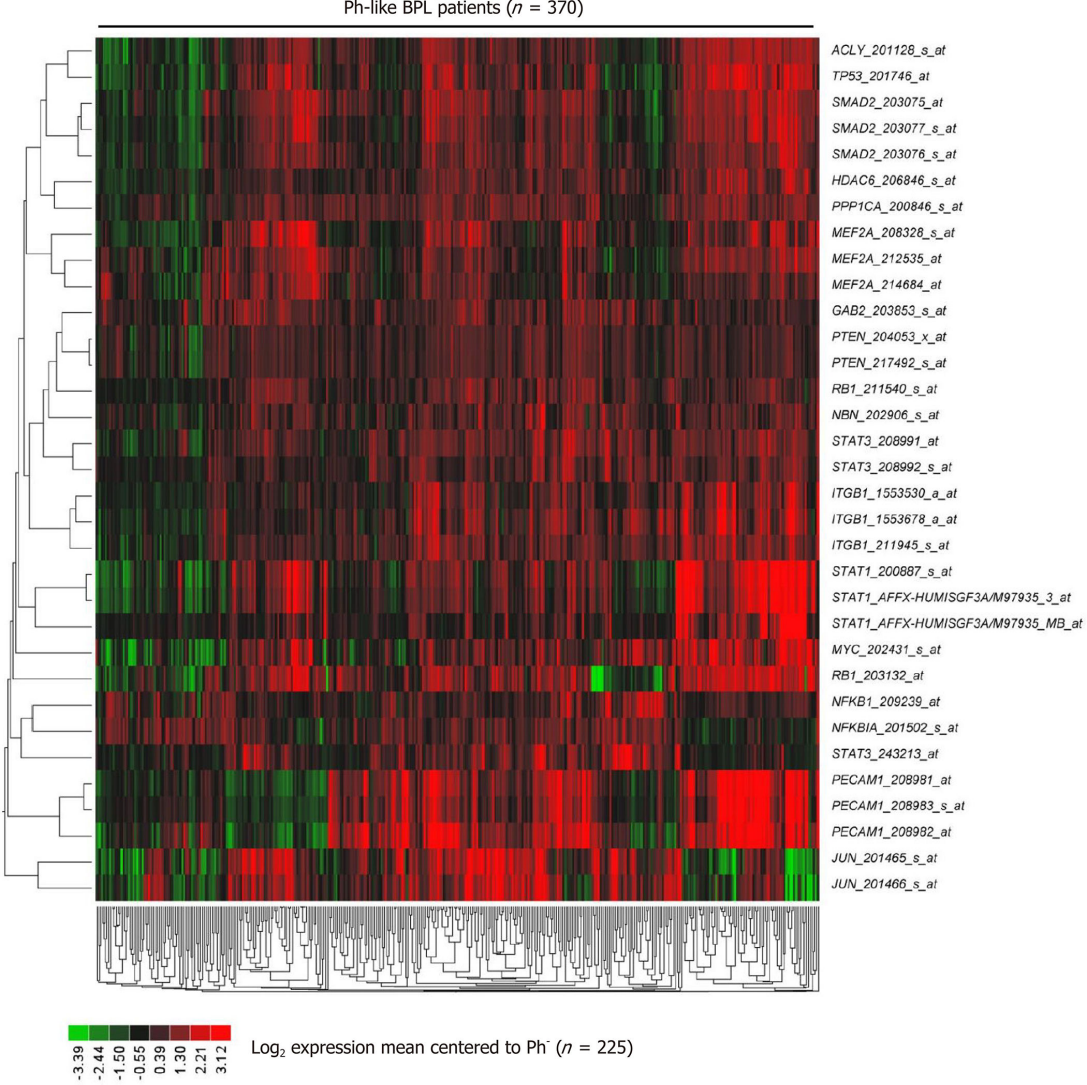
Expression of CD22ΔE12 signature genes in Ph-like ALL. In total 370 Ph-like ALL cases were identified using a set of 110 gene probeset gene expression signature developed utilizing the Affymetrix U133 plus 2.0 microarray platform, as described in the “METHODS”. The RMA-normalized gene expression values for leukemia cells obtained from these 370 Ph-like ALL patients [GSE11877 (*n* = 99), GSE13159 (*n* = 216), GSE13351(*n* = 55)] were log_2_ transformed and mean-centered to the average value for leukemia cells from 225 BCR-ABL^−^ BPL patients (GSE1187, GSE13159, GSE13351; Ph-like ALL, MLL-R^+^ and t[1;19]/E2A-PBX1^+^patients excluded). To determine the differential expression of each of the leading-edge genes of the CD22ΔE12 transcriptome, linear contrasts were performed for the mean-centered values. Heat map depicts up and down regulated transcripts for mean-centered expression values and clustered according to average distance metric

The signature transcriptome of CD22ΔE12-Tg mouse BPL cells is also a characteristic feature of primary leukemia cells from newly diagnosed Ph^+^ and Ph-like BPL patients as well as relapsed BPL patients. CD22ΔE12 is present in the vast majority of patients with Ph^+^ BPL as well as patients with Ph-like BPL [Figure 2]. Ph-like ALL cases were previously identified using a set of 110 gene probeset gene expression signature developed utilizing the Affymetrix U133 plus 2.0 microarray platform, as described in the Methods. In Figure 2A, the gene expression of each sample was correlated against the 110 probeset expression template resulting in a distribution of correlation coefficient values (*r*) for each subtype of BPL. Ph-like ALL cases were defined from a cut off “*r*” value such that the sample from the “*r*” value was greater than the 99% percentile of the “*r*” values in non-leukemic normal control samples and greater than the minimum “*r*” value for the known Ph^+^ ALL cases. Only B-precursor ALL (BPL) were included in these comparisons. In Figure 2B, the correlation method identified 370 Ph-like ALL patients whose leukemia cells had transcriptomes that were significantly different from the transcriptomes of normal hematopoietic cells of the control group (*n* = 74) (Non-parametric Wilcoxon test, *P* < 0.0001) with regards to correlation coefficient of the 110 probeset signature. The median (MED) range and *P*-value for comparison with the normal control group are reported for each of the ALL subsets. One hundred and five (85%) of Ph^+^ ALL cases and 342 (92%) of Ph-like ALL cases had CD22ΔE12-index values that were lower than the 95% lower confidence interval (CI) for the CD22ΔE12-index values for the 74 normal bone marrow samples (95% CI = 0.394–0.655) (planned linear contrasts, *P* < 0.0001). The average CD22ΔE12 index values were −0.07 ± 0.05 for the Ph^+^ ALL samples and −0.33 ± 0.03 for the Ph-like ALL samples. Only BPL patients were included in these comparisons. Likewise, there was a very high incidence of CD22ΔE12 in relapsed BPL^[8]^. Examination of this signature transcriptome in 123 BCR-ABL/Ph^+^ BPL patients showed that 36 genes represented by 60 probesets that were differentially expressed in 123 BCR-ABL^+^ patients with *P* < 0.0001 [Figure 3] of which 6 transcripts (*PECAM1*_208982_at, *PECAM1*_208981_at, *PECAM1*_208983_s_at, *ITGB1*_211945_s_at, *ITGB1*_1553678_a_at and *PAK1*_226507_at) were up regulated greater than 2 fold compared to other ALL subtypes (Fold difference ranged from 2.01 to 3.64 and *P*-values were less than 1.0 × 10^−16^ for these transcripts) [Supplementary Table 3]. Significantly over expressed signature transcriptome encoding the upregulated phosphoproteome in CD22ΔE12 KI mice revealed that 18 genes represented by 33 probesets were differentially expressed in 370 Ph-like BPL patients with *P* < 0.0001 [Figure 4]. Most significantly affected transcripts included *PECAM1*_208982_at, *SMAD2*_203075_at, *PECAM1*_208981_at, *PPP1CA*_200846_s_at, *PECAM1*_208983_s_at, *RB1*_203132_at and *STAT3*_208991_at (Fold difference values ranged from 1.40 to 1.66 and *P*-values ranged from 5.0 × 10^−12^ to 1.0 × 10^−16^) [Supplementary Table 1].

**Figure 5.**
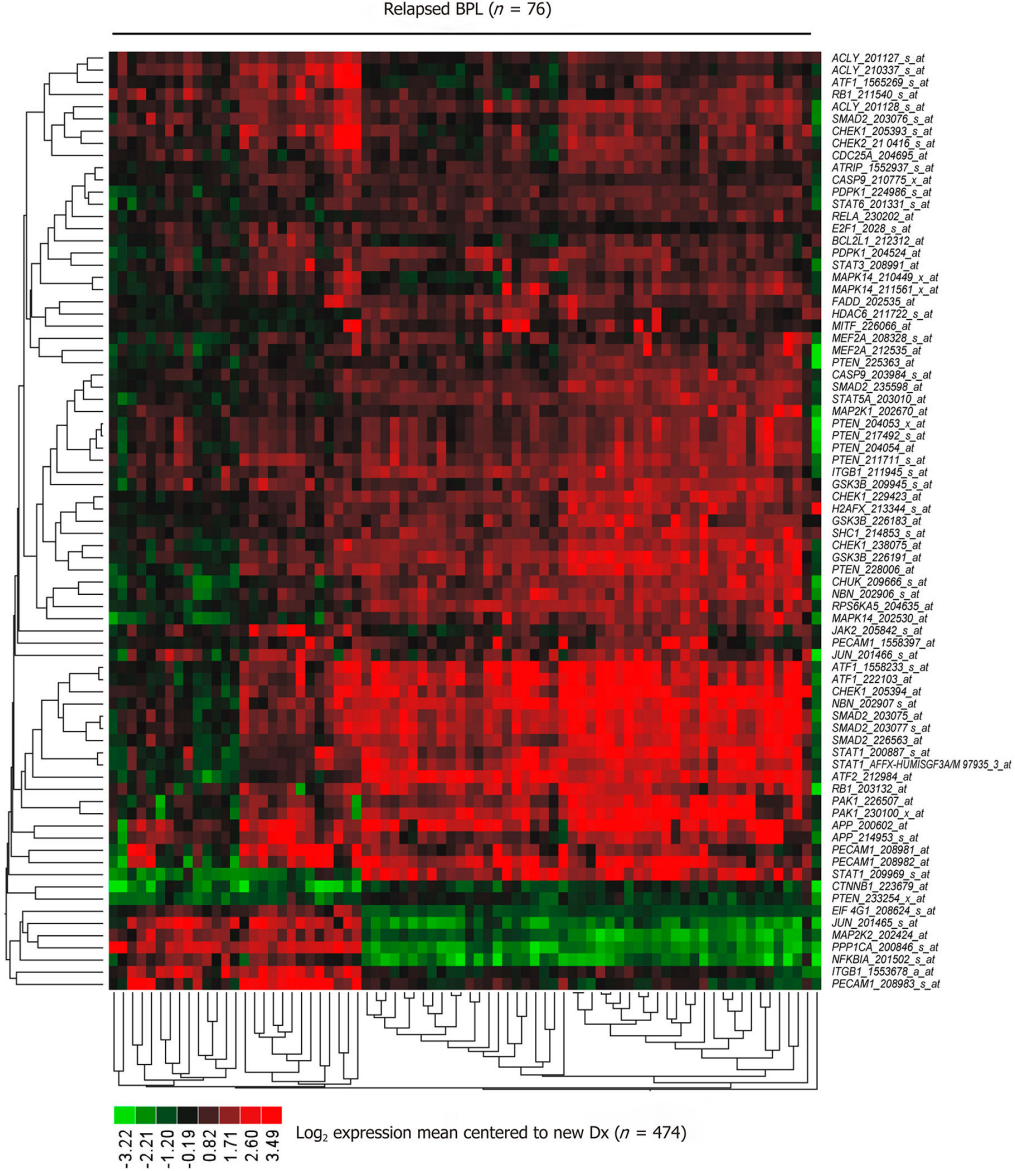
Representation of the CD22ΔE12 signature transcriptome in primary leukemia cells from relapsed BPL patients. The 57-gene mouse CD22ΔE12-KI transcriptome was represented by 183 probesets on the human U133 Plus 2.0 Array The RMA-normalized gene expression values for leukemia cells from 76 BPL patients in relapse (GSE28460, GSE18497) were log_2_ transformed and mean-centered to the average value for the 474 new diagnosis samples (GSE11877, GSE13351, GSE28460, GSE7440). Heat map depicts up and down regulated transcripts ranging from red to green respectively for expression values mean centered to diagnosis samples and clustered according to average distance metric. Forty-two signature genes represented by 77 probesets that were differentially expressed in 76 relapsed BPL patients with *P* < 0.0001 (37 genes represented by 70 probesets were upregulated). The cassette of 183 probesets were significantly up regulated in relapsed patients (Mixed Model ANOVA, F_1,548_ = 104, *P* < 0.00001) [Supplementary Table 2]

Comparing newly diagnosed patients with 76 relapsed patients resulted in 42 genes (77 probesets) being differentially expressed and 7 transcripts were up regulated greater than 3-fold (range = 3.06–3.83 increase, *P* < 1.0 × 10^−16^) in relapsed patients (*APP*_200602_at, *CHEK1*_205394_at, *NBN*_202907_s_at, *ATF1*_1558233_s_at, *SMAD2*_203077_s_at, *SMAD2*_203075_at, *ATF1*_222103_at) [Figure 5 and Supplementary Table 2].

Taken together, these results demonstrate that BPL cells from CD22ΔE12-Tg mice exhibit gene and protein expression profiles consistent with constitutive activation of multiple signaling networks [Figure 1]^[13]^, including the PI3-Kinase and MAP-Kinase pathways, mimicking the profiles of drug-resistant relapsed BPL patients as well as newly diagnosed patients with Ph^+^ BPL [Figure 3] as well as Ph-like BPL [Figure 4] Likewise, the CD22ΔE12 signature genes were differentially regulated in leukemia cells from relapsed BPL patients (*n* = 76) *vs*. newly diagnosed BPL patients (*n* = 474) [Figure 5]. CD22ΔE12 may therefore have clinical utility as a biomarker of aggressive relapse clones in high-risk BPL patients and a potential molecular target for more effective treatment of drug-resistant BPL.

### CD22ΔE12 as a unique molecular target for RNAi therapy

RNAi has emerged as an attractive technology for silencing the expression of specific genes in human cells. Nanoparticles represent particularly attractive delivery systems for small interfering RNA (siRNA) and may provide the foundation for rational design and formulation of RNAi-triggering nanomedicines. A more rapid development of nanoscale RNAi therapeutics has been hampered by the limited knowledge regarding the identity of the critical driver lesions in specific types of cancer, safety concerns about certain formulations, and a disappointing poor delivery of the siRNA into target cancer cells. CD22ΔE12 was recently discovered by our team as an ideal molecular target for RNAi therapy against relapsed BPL^[10,11,14]^. We first designed a phosphorothioate-modified bioactive siRNA duplex that specifically targets the Exon 11/Exon 13 junction of the CD22ΔE12 mutant mRNA unique to leukemia cells^[10,11,14]^. Transfection with CD22ΔE12 siRNA, but not scr-siRNA, caused selective CD22ΔE12 depletion in aggressive BPL xenograft cells, which was associated with a marked inhibition of their clonogenicity *in vitro*. The documented profound impact of modest-moderate CD22ΔE12 depletion on clonogenicity of these “difficult-to-transfect” cells indicates that clonogenic ALL cells are very dependent on CD22ΔE12 abundance^[10,11,14]^.

**Figure 6.**
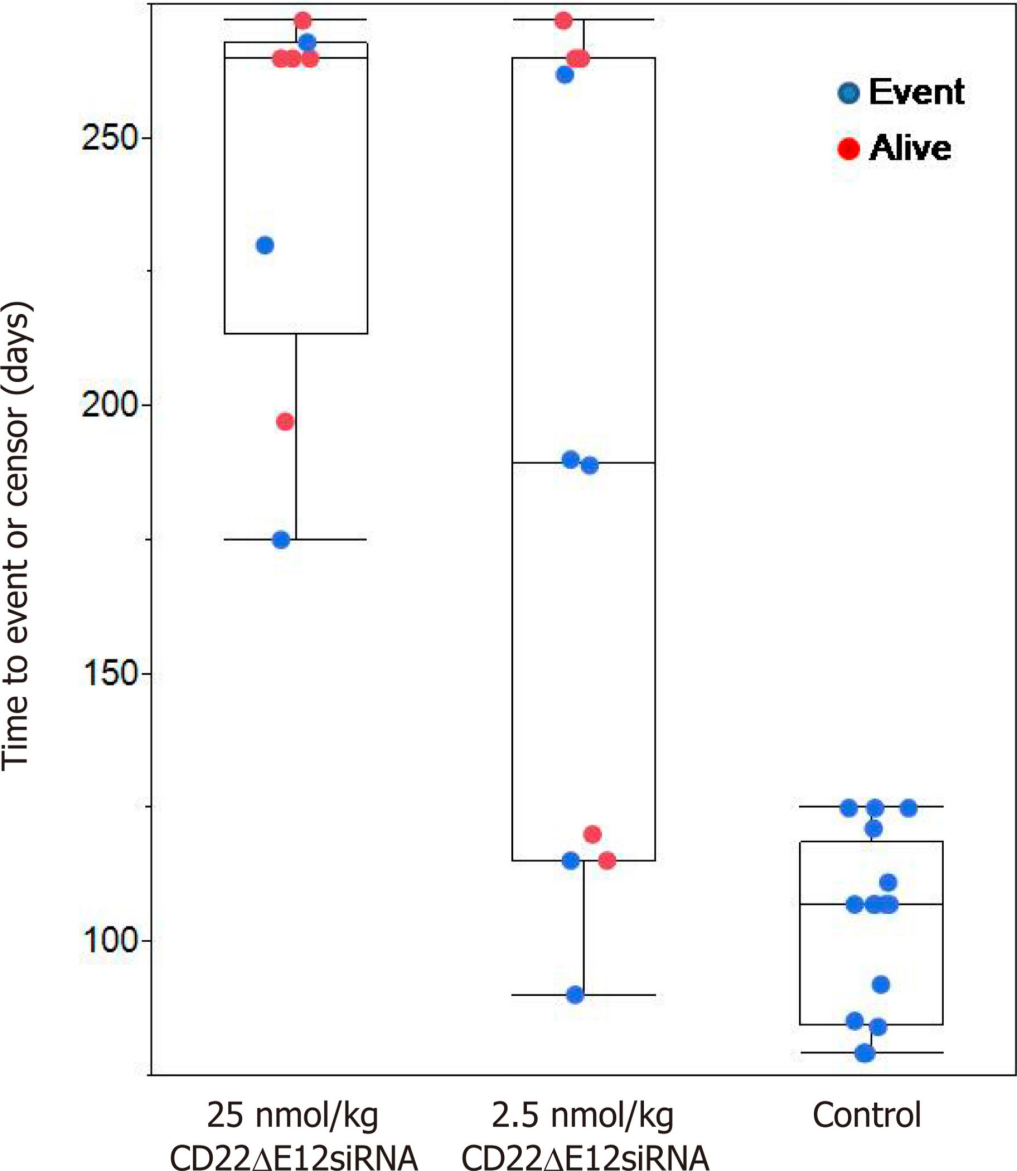
CD22ΔE12-siRNA liposomal nanoformulation exhibits potent *in vivo* anti-leukemic activity in NOD/SCID mouse xenograft models of relapsed human BPL. Mice were inoculated i.v. with xenograft cells [(Xeno Case #’s 5 and 10); 4 × 10^5^ cells/mouse] derived from primary leukemia cells of two pediatric patients with relapsed BPL. Sixteen control mice were either left untreated or treated with the liposomal control nanoformulation of scr-siRNA (25 nmol/kg/day × 3 days, day 1–3) or an empty control LNF. Test mice were treated with the LNF of CD22ΔE12-siRNA LNF (low-dose regimen = 2.5 nmol/kg/day × 3 days, day 1–3, *n* = 10; high-dose regimen = 25 nmols/kg/day × 3 days, day 1–3, *n* = 9). All 16 control treated mice died and 10 out of 19 CD22ΔE12 siRNA treated mice (pooled both concentrations) were censored alive during the study (Fisher’s exact test, 2 tailed, *P* = 0.0005). CD22ΔE12 treated displayed signifcantly longer time to event observations compared to control mice (median = 190 days for CD22ΔE12 treated *vs*. median = 107 days for control, Wilcoxon test, one-way Chi-square approximation, *P* = 0.0012)

In order to achieve RNAi in all leukemic cells in a targeted BPL cell population, we prepared a nanoscale liposomal formulation of CD22ΔE12 siRNA using a mixture of the cationic lipid DOTAP for complexation with the polyanionic siRNA cargo as well as cell membrane penetration and the helper neutral lipid DOPE^[11]^. CD22ΔE12-siRNA LNF abrogated the *in vivo* clonogenicity of the leukemia-initiating leukemic cell fraction in recently reported xenograft specimens^[11]^ derived from patients with relapsed BPL. Despite the shortcomings inherent to the PK of LNF, the CD22ΔE12-siRNA LNF significantly improved the EFS outcome of NS mice challenged with human BPL xenograft cells [Figure 6]^[11]^.

## DISCUSSION

The expression of CD22 on leukemic B-cell precursors has motivated the development and clinical testing of CD22-directed MoAb, recombinant fusion toxins and antibody-drug conjugates as therapeutic agents against BPL in children. However, these therapeutic modalities all target the surface epitopes of CD22 and do not discriminate between normal B-cells expressing intact CD22 and BPL cells expressing CD22ΔE12. Due to the presence of CD22 on normal human B-cells and B-cell precursors, lymphotoxicity with reduced B-cell numbers and possible hypogammaglobulinemia with an increased risk of infections would be anticipated side effects of CD22 directed MoAb and MoAb based therapeutics in clinical settings. In contrast, RNAi therapeutics targeting CD22ΔE12 would only kill BPL cells while leaving normal B-cell precursors and B-cells with an intact CD22 encoded by a wildtype CD22 mRNA unharmed.

The clinical development of personalized nanomedicines against the oncogenic driver lesion CD22ΔE12 in aggressive B-lineage leukemias and lymphomas would address an unmet and urgent challenge in the treatment of B-precursor acute lymphoblastic leukemia (BPL), the most common form of cancer in children and adolescents, and a leading cause of cancer - related deaths in children. Our overarching objective is the design of an innovative and effective new treatment strategy using an RNAi therapeutic targeting CD22ΔE12 to disrupt the constitutively active signaling networks that confer survival and proliferative advantages on CD22ΔE12^+^ BPL cells. The CD22-RNAi technology is applicable to all BPL patients both high risk and standard risk. That is because CD22ΔE12 is a characteristic feature of leukemic clones that escape chemotherapy and cause relapse in both high risk and low risk subgroups of patients. The technology therefore has the potential (1) for prevention of relapses by selectively killing the clones that are most likely to escape chemotherapy and cause relapse as well (2) for treatment of relapses in ALL. This research project may also lead to innovative 2nd line regimens against other forms of CD22ΔE12-positive relapsed B-lineage leukemias and lymphomas, including mantle cell lymphoma/leukemia, Burkitt’s lymphoma/leukemia, and diffuse large B-cell lymphoma.

Nanoparticles represent particularly attractive delivery systems for siRNA and may provide the foundation for rational design and formulation of RNAi-triggering nanomedicines. Our team has recently developed a highly efficient small interfering RNA (siRNA) delivery platform based on a rationally designed cell-penetrating cationic helical polypeptide. We are developing advanced multifunctional bioactive nanomaterials with optimized properties as siRNA delivery vehicles in an attempt to further improve the potency and broaden the therapeutic window of their nanocomplexes with therapeutic siRNA. We have reported membrane-penetrating endosomolytic hybrid nanocarriers for RNA interference therapy in poor prognosis BPL^[14]^. These novel nanomaterials exhibit unprecedented thermodynamic and physicochemical stability and unique biological properties with exceptional endosomal escape and nucleic acid delivery efficiency. Our nanoscale siRNA delivery platform has been further optimized to enhance its translational impact potential^[15–17]^. We have complexed CD22ΔE12-siRNA with a 200-mer polymer of the lead helical polypeptide to prepare a nanoscale formulation of CD22ΔE12-siRNA. This unique nanoparticle formulation caused marked CD22ΔE12 mRNA and protein depletion in BPL cells and inhibited their clonogenic growth. These hybrid nanocarrriers are being functionalized with a leukemia targeting moiety directed against the CD19 surface receptor on BPL cells in order to achieve optimal delivery to and uptake by BPL cells to further reduce their potential toxicity and improve their efficacy. Polypeptide-based siRNA nanocomplexes with CD19-binding functionality could represent an important addition to the emerging new personalized treatment options for B-lineage lymphoid malignancies.

CD19 is a 95-kDa B-lineage restricted receptor molecule that is expressed on leukemia cells from virtually 100% of BPL cases, but it is absent on the parenchymal cells of life-maintaining non-hematopoietic organs, circulating blood myeloid and erythroid cells, T-cells as well as bone marrow stem cells. The favorable leukemic cell *vs*. normal tissue expression profile of CD19 and its abundant expression on relapse BPL clones make it an attractive molecular target for biotherapy in relapsed ALL^[18]^. We recently cloned the gene encoding a natural ligand of the human CD19 receptor (CD19-L) from a human thymus cDNA library by yeast two-hybrid screening using cDNA encoding the human CD19 extracellular domain (AA 1 to 273) (CD19ECD), fused to the GAL4 DNA binding domain, as the bait plasmid^[19,20]^. We produced a 54-kDa soluble recombinant human CD19-L protein and confirmed its selective immunoreactivity with the extracellular domain of the human CD19 receptor^[19,20]^. Recombinant human CD19-L provides a unique opportunity to generate targeted NP by conjugating it onto the side chain of the PEGylated PVBLG-8 derivative. Thiol-maleimide conjugation will yield a stable thioether linkage between CD19L and PVBLG-8. Most importantly, we set out to prepare targeted nanoparticles by modifying the PVBLG-8 side chains with recombinant human CD19-L to optimize our lead nanoparticle formulation of CD22ΔE12-siRNA in an attempt to further improve its potency and broaden its therapeutic window. We hypothesize that siRNA nanocomplexes prepared using these 2nd generation nanomaterials with reconfigured PVBLG-8 building blocks will exhibit unprecedented *in vivo* RNAi potency owing to improved serum stability, pharmacokinetic properties, biodistribution and cellular uptake^[21]^. We do not anticipate any technical difficulties, but we are cognizant of the fact that the CD19-L-functionalized, CD19-directed nanoparticles may not necessarily prove more potent than untargeted siRNA nanoparticles. However, we anticipate that the PEGylation as well as CD19-L-grafting will favorably affect the *in vivo* stability of nanoparticles.

## Supplementary Material

SupplementaryTable1

SupplementaryTable2

SupplementaryTable3
