# Supplementary material for: Identification and targeting of CD22ΔE12 as a molecular RNAi target to overcome drug resistance in high-risk B-lineage leukemias and lymphomas
Source: Cancer Drug Resist. Author manuscript; Available in PMC 2019 Nov 29. (PMC6883925; doi:10.20517/cdr.2017.03)
Supplement: SupplementaryTable2 [file NIHMS1059614-supplement-SupplementaryTable2.pdf]

Supplementary Table 2: Representation of the CD22ΔE12 signature transcriptome in primary leukemia cells from relapsed BPL patients. The RMA-normalized gene expression values for leukemia cells from 76 BPL patients in relapse were log_2 transformed and mean-centered to the average value for the 474 new diagnosis samples Mixed Model ANOVA showed significant overall increase in expression of 110 probesets in relapsed cases ($F_{1,494} = 68.7$, $P < 0.0001$)[16]

Transcript	Fold difference (relapse *vs.* NewDx)	Linear contrast *P*-value
APP_200602_at	3.83	1.0E-16
CHEK1_205394_at	3.46	1.0E-16
NBN_202907_s_at	3.41	1.0E-16
ATF1_1558233_s_at	3.20	1.0E-16
SMAD2_203077_s_at	3.19	1.0E-16
SMAD2_203075_at	3.18	1.0E-16
ATF1_222103_at	3.06	1.0E-16
PAK1_230100_x_at	2.97	1.0E-16
PECAM1_208982_at	2.96	1.0E-16
ATF2_212984_at	2.77	1.0E-16
STAT1_200887_s_at	2.76	1.0E-16
STAT1_AFFX-HUMISGF3A/M97935_3_at	2.69	1.0E-16
SMAD2_226563_at	2.61	1.0E-16
GSK3B_226191_at	2.60	1.0E-16
RB1_203132_at	2.53	1.0E-16
PTEN_211711_s_at	2.38	1.0E-16
APP_214953_s_at	2.37	1.0E-16

PTEN_228006_at	2.36	1.0E-16
PAK1_226507_at	2.32	1.0E-16
ITGB1_211945_s_at	2.27	1.0E-16
PECAM1_208981_at	2.25	1.0E-16
CHEK1_238075_at	2.23	1.0E-16
CHEK1_229423_at	2.22	1.0E-16
GSK3B_209945_s_at	2.22	1.0E-16
ACLY_201128_s_at	2.20	1.0E-16
SHC1_214853_s_at	2.15	1.0E-16
NBN_202906_s_at	2.10	1.0E-16
H2AFX_213344_s_at	2.02	1.0E-16
RPS6KA5_204635_at	2.01	1.0E-16
GSK3B_226183_at	1.96	1.0E-16
CHEK1_205393_s_at	1.94	1.0E-16
PTEN_217492_s_at	1.92	1.0E-16
PTEN_204053_x_at	1.91	1.0E-16
STAT1_209969_s_at	1.90	1.0E-16
PTEN_204054_at	1.90	1.0E-16
SMAD2_235598_at	1.89	1.0E-16
SMAD2_203076_s_at	1.85	1.0E-16
JUN_201466_s_at	1.84	1.0E-16
RB1_211540_s_at	1.82	1.0E-16
CHEK2_210416_s_at	1.80	4.4E-16
STAT5A_203010_at	1.78	1.3E-15
CASP9_203984_s_at	1.74	1.7E-14

STAT3_208991_at	1.73	2.0E-14
PDPK1_204524_at	1.73	2.6E-14
ACLY_201127_s_at	1.71	9.9E-14
MAP2K1_202670_at	1.68	6.8E-13
CHUK_209666_s_at	1.67	8.7E-13
FADD_202535_at	1.67	9.6E-13
PECAM1_1558397_at	1.61	4.6E-11
MAPK14_211561_x_at	1.60	5.0E-11
JAK2_205842_s_at	1.60	5.1E-11
CDC25A_204695_at	1.60	5.6E-11
ATF1_1565269_s_at	1.60	7.2E-11
ITGB1_1553678_a_at	1.56	8.6E-10
ACLY_210337_s_at	1.55	1.2E-09
PECAM1_208983_s_at	1.51	8.5E-09
STAT6_201331_s_at	1.51	1.3E-08
MAPK14_202530_at	1.49	2.5E-08
CASP9_210775_x_at	1.49	2.6E-08
MEF2A_208328_s_at	1.48	6.4E-08
ATRIP_1552937_s_at	1.44	3.7E-07
PDPK1_224986_s_at	1.43	6.6E-07
HDAC6_211722_s_at	1.42	9.3E-07
MEF2A_212535_at	1.42	1.3E-06
MITF_226066_at	1.40	2.4E-06
E2F1_2028_s_at	1.37	1.3E-05
BCL2L1_212312_at	1.35	2.9E-05

PTEN_225363_at	1.35	3.4E-05
RELA_230202_at	1.35	3.6E-05
MAPK14_210449_x_at	1.34	5.2E-05
NBN_217299_s_at	1.31	2.0E-04
JAK2_205841_at	1.30	2.2E-04
BCL2L1_206665_s_at	1.30	2.3E-04
NFKBIE_203927_at	1.29	3.6E-04
BAD_209364_at	1.29	4.1E-04
PTEN_227469_at	1.28	5.2E-04
SHC1_201469_s_at	1.28	5.8E-04
STAT3_243213_at	1.28	6.4E-04
RPS6KA5_204633_s_at	1.27	8.1E-04
ESR1_205225_at	1.26	1.2E-03
TP53_211300_s_at	1.26	1.3E-03
H2AFX_205436_s_at	1.25	1.8E-03
SRC_1558211_s_at	1.25	2.0E-03
ESR1_217190_x_at	1.25	2.1E-03
MAPK14_211087_x_at	1.25	2.2E-03
TREX1_205875_s_at	1.24	2.7E-03
STAT1_AFFX-HUMISGF3A/M97935_MB_at	1.21	7.2E-03
DAXX_201763_s_at	1.21	7.4E-03
KCNIP3_228269_x_at	1.21	8.9E-03
MYC_202431_s_at	1.20	9.7E-03
STAT3_225289_at	1.19	1.8E-02

MAP2K2_213487_at	1.18	2.2E-02
E2F1_204947_at	1.17	2.5E-02
TP53_201746_at	1.17	3.4E-02
SRF_202401_s_at	1.16	3.8E-02
ESR1_217163_at	1.16	3.9E-02
STAT3_208992_s_at	1.14	6.7E-02
GSK3B_240562_at	1.14	7.2E-02
ESR1_215551_at	1.14	7.6E-02
KCNIP3_1555694_a_at	1.13	8.2E-02
GSK3B_242336_at	1.13	8.2E-02
DAXX_216038_x_at	1.13	8.6E-02
NBN_202905_x_at	1.13	8.8E-02
CDC25A_1555772_a_at	1.12	1.1E-01
STAT1_AFFX-HUMISGF3A/M97935_MA_at	1.12	1.2E-01
CASP9_240437_at	1.12	1.2E-01
MEF2A_214684_at	1.12	1.2E-01
MITF_207233_s_at	1.11	1.4E-01
H2AFX_212524_x_at	1.10	1.9E-01
HDAC6_216224_s_at	1.10	2.0E-01
NFKBIA_231699_at	1.10	2.0E-01
PDPK1_32029_at	1.09	2.1E-01
JUN_201464_x_at	1.09	2.2E-01
ESR1_211233_x_at	1.08	2.6E-01
ITGB1_1553530_a_at	1.08	2.7E-01

NBN_240510_at	1.08	2.8E-01
SRC_213324_at	1.08	3.0E-01
JUN_213281_at	1.08	3.1E-01
RELA_201783_s_at	1.07	3.2E-01
TREX1_34689_at	1.07	3.3E-01
SMAD2_239271_at	1.07	3.3E-01
BCL2L1_231228_at	1.07	3.3E-01
GAB2_203853_s_at	1.07	3.7E-01
PDPK1_244629_s_at	1.06	4.0E-01
BCL2L1_215037_s_at	1.06	4.3E-01
SRF_202400_s_at	1.06	4.3E-01
GATA1_1555590_a_at	1.05	4.6E-01
PDPK1_244630_at	1.05	4.6E-01
BAD_1861_at	1.05	4.6E-01
PDPK1_232050_at	1.05	4.6E-01
PPARG_208510_s_at	1.05	4.7E-01
ESR1_211235_s_at	1.05	4.7E-01
KCNIP3_231774_at	1.05	5.2E-01
ESR1_211234_x_at	1.04	5.9E-01
ESR1_215552_s_at	1.04	6.0E-01
GATA1_210446_at	1.04	6.1E-01
KDR_203934_at	1.03	6.6E-01
SRC_221281_at	1.03	6.7E-01
RPS6KA5_1554319_at	1.02	7.9E-01
PAK1_1565772_at	1.02	8.0E-01

HDAC6_206846_s_at	1.02	8.0E-01
SRC_1565082_x_at	1.02	8.3E-01
ITGB1_216190_x_at	1.01	8.5E-01
PAK1_209615_s_at	1.01	8.5E-01
PECAM1_1559921_at	1.00	9.5E-01
CTNNB1_1554411_at	1.00	9.6E-01
CDC25A_204696_s_at	1.00	9.7E-01
PDPK1_221244_s_at	1.00	9.9E-01
EIF4G1_208625_s_at	1.00	9.8E-01
KCNIP3_233688_at	1.00	9.6E-01
MITF_1554874_at	1.00	9.5E-01
KCNIP3_1569355_at	0.99	8.6E-01
STAT1_AFFX-HUMISGF3A/M97935_5_at	0.98	7.7E-01
SRC_1558210_at	0.98	7.5E-01
KCNIP3_234647_at	0.97	7.0E-01
ITGB1_216178_x_at	0.96	5.6E-01
ITGB1_215879_at	0.96	5.4E-01
MAP2K2_213490_s_at	0.95	4.8E-01
BAD_232660_at	0.94	3.9E-01
SRC_221284_s_at	0.94	3.9E-01
ITGB1_215878_at	0.94	3.8E-01
SRC_1565080_at	0.93	3.4E-01
STAT6_201332_s_at	0.93	3.1E-01
MEF2A_242176_at	0.92	2.6E-01
VASP_202205_at	0.92	2.3E-01

KCNIP3_234198_at	0.92	2.3E-01
ESR1_211627_x_at	0.91	1.7E-01
ATF2_1555146_at	0.89	1.1E-01
APP_211277_x_at	0.86	3.0E-02
RELA_209878_s_at	0.84	1.3E-02
ATF2_205446_s_at	0.82	6.5E-03
CTNNB1_201533_at	0.82	5.9E-03
PTEN_242622_x_at	0.82	4.8E-03
JAK2_1562031_at	0.81	4.0E-03
H2AFX_212525_s_at	0.80	1.8E-03
NFKB1_209239_at	0.78	4.9E-04
EIF4G1_208624_s_at	0.73	1.4E-05
JUN_201465_s_at	0.70	5.7E-07
PPP1CA_200846_s_at	0.67	3.3E-08
MAP2K2_202424_at	0.63	2.3E-10
PTEN_233254_x_at	0.59	5.3E-13
CTNNB1_223679_at	0.47	1.0E-16
NFKBIA_201502_s_at	0.46	1.0E-16
