# Supplementary material for: Identification and targeting of CD22ΔE12 as a molecular RNAi target to overcome drug resistance in high-risk B-lineage leukemias and lymphomas
Source: Cancer Drug Resist. Author manuscript; Available in PMC 2019 Nov 29. (PMC6883925; doi:10.20517/cdr.2017.03)
Supplement: SupplementaryTable3 [file NIHMS1059614-supplement-SupplementaryTable3.pdf]

Supplementary Table 3: Expression of CD22ΔE12 signature genes in Ph$^+$/BCR-ABL$^+$ BPL. The RMA-normalized gene expression values for leukemia cells obtained from 123 BCR-ABL$^+$ patients were log_2-transformed and mean-centered to the average value for leukemia cells from 225 BCR-ABL$^-$ BPL patients (Ph-like ALL, MLL-R$^+$ and t[1;19]/E2A-PBX1$^+$ patients excluded). To determine the differential expression of each of the leading-edge genes of the CD22ΔE12 transcriptome, linear contrasts were performed for the mean-centered values

**Transcript**	**Fold difference (BCR-ABL$^+$ *vs.* other)**	**Linear contrast *P*-value**
PECAM1_208982_at	3.64	1.0E-16
PECAM1_208981_at	2.98	1.0E-16
PECAM1_208983_s_at	2.49	1.0E-16
ITGB1_211945_s_at	2.13	1.0E-16
ITGB1_1553678_a_at	2.07	1.0E-16
PAK1_226507_at	2.01	1.0E-16
JUN_201465_s_at	1.94	1.0E-16
STAT3_208991_at	1.85	1.0E-16
NBN_202907_s_at	1.82	1.0E-16
PAK1_230100_x_at	1.75	1.0E-16
PPP1CA_200846_s_at	1.72	1.0E-16
NBN_202906_s_at	1.71	1.0E-16
MAP2K2_202424_at	1.70	2.2E-16
ITGB1_1553530_a_at	1.69	8.9E-16
BCL2L1_212312_at	1.69	8.9E-16
STAT3_225289_at	1.67	2.7E-15
NBN_217299_s_at	1.67	3.1E-15

RB1_211540_s_at	1.64	2.9E-14
PDPK1_204524_at	1.63	6.6E-14
NBN_202905_x_at	1.62	7.6E-14
STAT6_201331_s_at	1.61	2.0E-13
PECAM1_1558397_at	1.61	2.1E-13
SHC1_214853_s_at	1.59	1.0E-12
VASP_202205_at	1.57	3.8E-12
APP_200602_at	1.56	5.7E-12
STAT1_200887_s_at	1.51	1.6E-10
SMAD2_203075_at	1.51	1.8E-10
MYC_202431_s_at	1.49	6.0E-10
STAT5A_203010_at	1.49	6.2E-10
MAP2K2_213490_s_at	1.47	2.1E-09
JUN_201466_s_at	1.47	3.3E-09
PAK1_209615_s_at	1.43	3.1E-08
STAT3_243213_at	1.43	3.4E-08
RB1_203132_at	1.43	3.4E-08
EIF4G1_208624_s_at	1.43	4.6E-08
STAT3_208992_s_at	1.42	6.0E-08
GSK3B_209945_s_at	1.41	1.1E-07
SMAD2_203076_s_at	1.40	2.5E-07
MAP2K1_202670_at	1.39	2.9E-07
ACLY_201128_s_at	1.39	3.7E-07
BAD_209364_at	1.39	4.7E-07
STAT1_AFFX-HUMISGF3A/M97935_3_at	1.39	4.7E-07

HDAC6_206846_s_at	1.39	4.9E-07
MEF2A_212535_at	1.38	8.5E-07
PTEN_228006_at	1.37	1.0E-06
NFKBIE_203927_at	1.37	1.0E-06
RELA_201783_s_at	1.36	1.6E-06
MEF2A_208328_s_at	1.36	2.2E-06
TREX1_205875_s_at	1.36	2.4E-06
PTEN_204054_at	1.35	4.6E-06
PTEN_204053_x_at	1.33	8.3E-06
TP53_201746_at	1.32	1.7E-05
BAD_1861_at	1.32	2.1E-05
NFKBIA_201502_s_at	1.31	3.3E-05
TREX1_34689_at	1.31	3.8E-05
NFKB1_209239_at	1.30	4.5E-05
PTEN_217492_s_at	1.30	5.5E-05
GAB2_203853_s_at	1.29	7.4E-05
STAT1_209969_s_at	1.28	1.5E-04
ATF2_212984_at	1.28	1.5E-04
JAK2_1562031_at	1.28	1.7E-04
PTEN_211711_s_at	1.26	2.9E-04
JAK2_205842_s_at	1.25	4.9E-04
MAPK14_202530_at	1.25	6.4E-04
CASP9_203984_s_at	1.25	6.5E-04
GSK3B_226191_at	1.24	7.3E-04
SMAD2_203077_s_at	1.23	1.5E-03

EIF4G1_208625_s_at	1.22	2.5E-03
ATF1_1565269_s_at	1.20	4.1E-03
JUN_201464_x_at	1.20	4.9E-03
PDPK1_224986_s_at	1.20	5.4E-03
H2AFX_205436_s_at	1.19	7.8E-03
ATF1_1558233_s_at	1.18	9.5E-03
ATF1_222103_at	1.18	1.2E-02
CASP9_210775_x_at	1.16	2.6E-02
ATF2_205446_s_at	1.15	2.7E-02
PTEN_242622_x_at	1.15	2.8E-02
JAK2_205841_at	1.15	2.8E-02
PAK1_1565772_at	1.15	3.3E-02
APP_214953_s_at	1.14	4.4E-02
STAT6_201332_s_at	1.13	5.7E-02
PTEN_225363_at	1.13	6.1E-02
PDPK1_32029_at	1.12	7.2E-02
ATRIP_1552937_s_at	1.12	7.5E-02
RPS6KA5_204633_s_at	1.11	1.1E-01
SHC1_201469_s_at	1.10	1.3E-01
PECAM1_1559921_at	1.10	1.3E-01
ACLY_210337_s_at	1.10	1.3E-01
MAPK14_211561_x_at	1.10	1.5E-01
GATA1_210446_at	1.10	1.6E-01
ESR1_211234_x_at	1.09	1.7E-01
ACLY_201127_s_at	1.09	1.8E-01

GATA1_1555590_a_at	1.09	1.9E-01
BAD_232660_at	1.08	2.2E-01
BCL2L1_231228_at	1.08	2.3E-01
SRC_221281_at	1.08	2.3E-01
DAXX_201763_s_at	1.08	2.3E-01
SMAD2_226563_at	1.08	2.4E-01
KCNIP3_231774_at	1.08	2.6E-01
CTNNB1_201533_at	1.08	2.6E-01
ITGB1_216178_x_at	1.07	2.7E-01
RELA_209878_s_at	1.07	2.7E-01
ESR1_211233_x_at	1.07	2.9E-01
STAT1_AFFX-HUMISGF3A/M97935_MB_at	1.07	2.9E-01
PTEN_233254_x_at	1.06	3.5E-01
KCNIP3_234198_at	1.06	3.5E-01
RPS6KA5_204635_at	1.06	3.6E-01
FADD_202535_at	1.06	3.7E-01
NBN_240510_at	1.06	3.7E-01
MEF2A_214684_at	1.05	4.6E-01
KCNIP3_228269_x_at	1.05	4.6E-01
CHEK1_229423_at	1.05	4.7E-01
SMAD2_235598_at	1.05	5.0E-01
PDPK1_221244_s_at	1.04	5.1E-01
MAPK14_210449_x_at	1.04	5.2E-01
PTEN_227469_at	1.04	5.3E-01

ESR1_211627_x_at	1.04	5.8E-01
SRC_1558211_s_at	1.03	6.2E-01
GSK3B_240562_at	1.03	6.4E-01
HDAC6_216224_s_at	1.03	6.6E-01
SRF_202400_s_at	1.03	6.7E-01
TP53_211300_s_at	1.03	6.9E-01
PDPK1_244629_s_at	1.02	7.3E-01
KCNIP3_1555694_a_at	1.02	7.6E-01
RELA_230202_at	1.02	7.9E-01
ESR1_211235_s_at	1.02	8.1E-01
CHUK_209666_s_at	1.01	8.4E-01
KCNIP3_234647_at	1.01	8.6E-01
CTNNB1_1554411_at	1.01	8.8E-01
DAXX_216038_x_at	1.01	9.2E-01
H2AFX_212524_x_at	1.01	9.2E-01
H2AFX_213344_s_at	1.01	9.3E-01
SMAD2_239271_at	1.00	9.7E-01
CDC25A_204695_at	1.00	1.0E+00
GSK3B_226183_at	1.00	9.7E-01
H2AFX_212525_s_at	1.00	9.7E-01
SRC_1565080_at	1.00	9.4E-01
CASP9_240437_at	1.00	9.4E-01
MEF2A_242176_at	0.99	9.1E-01
SRC_221284_s_at	0.99	8.7E-01
ITGB1_215879_at	0.99	8.6E-01

PDPK1_232050_at	0.99	8.5E-01
ITGB1_215878_at	0.98	7.9E-01
ESR1_205225_at	0.98	7.8E-01
ESR1_217163_at	0.98	7.8E-01
KCNIP3_233688_at	0.98	7.6E-01
MAP2K2_213487_at	0.98	7.5E-01
ESR1_215552_s_at	0.98	7.4E-01
STAT1_AFFX-HUMISGF3A/M97935_MA_at	0.98	7.4E-01
KDR_203934_at	0.98	7.3E-01
STAT1_AFFX-HUMISGF3A/M97935_5_at	0.98	7.0E-01
SRF_202401_s_at	0.97	6.7E-01
NFKBIA_231699_at	0.97	6.6E-01
MAPK14_211087_x_at	0.97	6.6E-01
SRC_1565082_x_at	0.97	6.5E-01
MITF_1554874_at	0.97	6.3E-01
ESR1_215551_at	0.97	5.9E-01
MITF_207233_s_at	0.96	5.6E-01
SRC_213324_at	0.96	5.4E-01
BCL2L1_206665_s_at	0.96	4.9E-01
KCNIP3_1569355_at	0.96	4.9E-01
RPS6KA5_1554319_at	0.96	4.8E-01
SRC_1558210_at	0.95	4.6E-01
BCL2L1_215037_s_at	0.95	4.3E-01
ITGB1_216190_x_at	0.94	3.8E-01

PPARG_208510_s_at	0.94	3.2E-01
HDAC6_211722_s_at	0.94	3.1E-01
CHEK2_210416_s_at	0.93	2.9E-01
GSK3B_242336_at	0.93	2.5E-01
ESR1_217190_x_at	0.92	2.3E-01
ATF2_1555146_at	0.90	1.1E-01
E2F1_204947_at	0.89	7.6E-02
CDC25A_204696_s_at	0.89	6.9E-02
APP_211277_x_at	0.88	4.8E-02
CHEK1_205394_at	0.87	2.7E-02
CHEK1_205393_s_at	0.86	2.0E-02
PDPK1_244630_at	0.85	1.4E-02
E2F1_2028_s_at	0.85	1.1E-02
JUN_213281_at	0.84	6.9E-03
MITF_226066_at	0.81	1.4E-03
CHEK1_238075_at	0.79	2.7E-04
CTNNB1_223679_at	0.77	3.6E-05
CDC25A_1555772_a_at	0.76	3.2E-05
